# High-efficiency procedure to characterize, segment, and quantify complex multicellularity in raw micrographs in plants

**DOI:** 10.1186/s13007-020-00642-0

**Published:** 2020-07-28

**Authors:** Xi Zhang, Zijian Hu, Yayu Guo, Xiaoyi Shan, Xiaojuan Li, Jinxing Lin

**Affiliations:** 1 Beijing Advanced Innovation Center for Tree Breeding by Molecular Design, Beijing Forestry University, Beijing, 10083 China; 2 College of Biological Sciences and Biotechnology, Beijing Forestry University, Beijing, 10083 China

**Keywords:** Large-scale imaging, Plant multicellularity, Morphogenesis, Parameters, Image processing

## Abstract

**Background:**

The increasing number of novel approaches for large-scale, multi-dimensional imaging of cells has created an unprecedented opportunity to analyze plant morphogenesis. However, complex image processing, including identifying specific cells and quantitating parameters, and high running cost of some image analysis softwares remains challenging. Therefore, it is essential to develop an efficient method for identifying plant complex multicellularity in raw micrographs in plants.

**Results:**

Here, we developed a high-efficiency procedure to characterize, segment, and quantify plant multicellularity in various raw images using the open-source software packages ImageJ and SR-Tesseler. This procedure allows for the rapid, accurate, automatic quantification of cell patterns and organization at different scales, from large tissues down to the cellular level. We validated our method using different images captured from *Arabidopsis thaliana* roots and seeds and *Populus tremula* stems, including fluorescently labeled images, Micro-CT scans, and dyed sections. Finally, we determined the area, centroid coordinate, perimeter, and Feret’s diameter of the cells and harvested the cell distribution patterns from Voronoï diagrams by setting the threshold at localization density, mean distance, or area.

**Conclusions:**

This procedure can be used to determine the character and organization of multicellular plant tissues at high efficiency, including precise parameter identification and polygon-based segmentation of plant cells.

## Background

Plants derive multiple adaptive advantages from their complex multicellular structural organization [1], which includes intricate molecule interactions, vesicle transport, and cellular interactions [2–5]. Diverse cellular structures confer higher-order functionality to the entire plant system [6, 7]. Unfortunately, most of our present understanding of cellular biology in plants has been obtained by analyzing phenotypes at the population level, which likely masks the differences between cells. The development of optical microscopes, X-ray microscopes, and electron/ion microscopes has provided technical support for biological research from the macroscopic scale to the nanoscale. The increasing use of three-dimensional (3D) large-scale imaging techniques, including nuclear magnetic resonance imaging (NMRI), micro/nano-computed tomography (Micro/Nano-CT), light sheet fluorescence microscopy (LSFM), laser scanning confocal microscopy (LSCM), and volume electron microscopy (Volume EM) has facilitated research at the tissue or cellular level and has led to high-throughput production of images of numerous cellular systems [8–11]. Although cellular-level imaging helps uncover precise multicellular properties [12], it remains challenging to develop specific methods for characterizing global properties, assemblies, and connectivity from complex cellular configurations.

ImageJ, one of the most widely used open-source imaging packages, is a processing platform for multidimensional biological image data [13]. The various versions of ImageJ are compatible with a wide variety of computer systems. ImageJ has been successfully used for several types of cell biology analysis, including co-localization analysis, fluorescence intensity quantitation, and 3D image reconstruction. Various ImageJ plugins allow it to be used for high-throughput image analysis for accurate, rapid export of massive amounts of data [14]. For example, the Trainable Weka Segmentation (TWS) plugin is a machine-learning tool that is often used for automatic tissue segmentation [15]. TrakEM2 is a powerful plugin for morphological data mining, 3D modeling, and image stitching, registration, editing, and annotation [16]. In addition, digital image analysis can be used for chemical phase identification and particle size determination by analyzing particles in binary images with ImageJ [17]. The ability to perform rapid, accurate analysis of multicellular properties is desirable in various cell biology fields, providing rich data for developmental and system organization studies. For instance, we previously performed particle analysis to calculate the properties of endocytic dots [18–21].

In mathematics, the Voronoï diagram (named after Georgy Voronoi), also known as the Dirichlet tessellation (named after Lejeune Dirichlet) or Voronoï tessellation, is a group of contiguous polygons that are closely fitted together in a repeated pattern without gaps or overlaps [22, 23]. The Voronoï diagram, which contains discrete data points connected to a Delaunay triangle network, is a partition of a planar space; this partition is key to establishing the tessellation algorithm [24]. Centroidal Voronoï tessellation is a useful tool with applications in many fields ranging from geography, meteorology, and crystallography to the aerospace industry. This tool analyzes the nearest point in a structure, the minimum closed circle, and many spatial measurements including adjacency, proximity, and accessibility analysis [25–28]. In cell biology, VoronoÏ tessellations have been used to model the geometric arrangement of cells in morphogenetic or cancerous tissues [29]. The open-source SR-Tesseler segmentation software package based on Voronoï tessellation was recently developed for the precise, robust, automatic quantification of protein organization from single-molecule localization microscopy images [30–33]. SR-Tesseler can also be used to detect cell clustering based on the spatial distribution of cellular centroidal points. In addition, SR-Tesseler can be used to segment a dense multicellular structure by setting the threshold of these polygons at average localization densities, mean distance, and area, making it suitable for analyzing multicellularity in plants.

Here, we designed a cellular recognition and quantitation procedure based on ImageJ and SR-Tesseler software and used it to investigate multicellularity in plant tissues using raw microscopy images. By applying brightness/contrast adjustment, smooth/sharp processing, threshold/binary conversion, and erode/dilate functions to raw data, ImageJ can be used to optimize cellular outlines, after which the cellular properties are harvested. After converting centroid data into .cvs format, the automatic quantification of cellular organization and connectivity can be performed by presenting a segmentation framework based on Voronoï tessellation in SR-Tesseler. Our strategy has several advantages for determining cellular properties and organization, as it allows the efficient optimization of raw images and the global identification of cellular properties. Subsequently, the centroid of cells is segmented, generating polygonal diagrams with normalized averages of localization density, mean distance, and area. The results generated by our procedure include cell number, area, perimeter, Feret’s diameter, distribution, organization structure, connectivity, and their correlations, allowing researchers to evaluate the tradeoffs and homeostasis involved in plant morphogenesis and development.

## Results

**Fig. 1:**
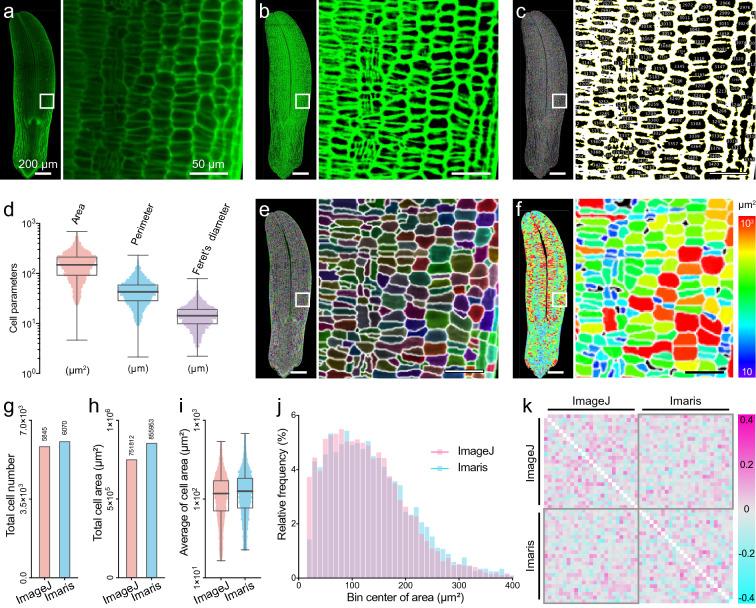
Recognition and qualification of *Populus trichocarpa* embryo cells by ImageJ and Imaris and their comparison. **a** Raw image of a *Populus trichocarpa* embryo captured by light sheet fluorescence microscopy (LSFM). **b** Compensation for the non-homogeneous fluorescent signal distribution in **a** using the ‘Plane Brightness Adjustment’ plugin. **c** Image of cell recognition and qualification by ImageJ software. **d** Quantification of cell area, perimeter, and Feret’s diameter from **c**. Boxplots represent mean, 25th, and 75th quartiles, whiskers represent minimum and maximum. n = 5845. **e** Image of cell recognition and qualification by Imaris software. **f** Heatmap of cell area calculated from **e**. The color scale represents the cell areas. **g**–**j** Comparison of values calculated by ImageJ and Imaris software. Statistical diagram of total cell number (**g**), total cell area (**h**), average cell area (**i**), and relative frequency of cell area. Boxplots represent mean, 25th, and 75th quartiles, whiskers represent minimum and maximum. n = 5845 and 6070. **k** Heatmap of correlation matrix analysis (Pearson, confidence interval = 95%) between cell areas (each cell area dataset is divided into 25 groups) calculated by ImageJ and Imaris software. In **a**–**c**, **e**, and **f**, panels on the right show enlarged images of areas on the left highlighted with white boxes; bar = 200 and 50 μm respectively

We used ImageJ to identify and quantify an image of a propidium iodide-labeled *Populus tremula* embryo captured by LSFM, which uncovered thousands of cellular structures (Fig. 1a). In general, the fluorescent signals from specimens created from deep cellular layers were weaker than those generated from the topmost layer due to the attenuation and distortion of the illumination light. We compensated for the non-homogeneous fluorescence signal using the ImageJ plugin ‘Plane Brightness Adjustment.jar’. The adjusted images showed much more uniform fluorescence compared to unadjusted images (Fig. 1b). After adjusting the contrast, brightness, and threshold, we identified and quantified the area, perimeter, and Feret’s diameter of the cells from the raw images (Fig. 1c, d).

To verify the results obtained by ImageJ, we analyzed the same raw image with Bitplane Imaris, a powerful software tool for 3/4D image visualization and analysis (Fig. 1e). In addition to segmenting cell outlines with Imaris, we generated a heatmap color-coded according to cell area (Fig. 1f). The total cell numbers and cell areas acquired by ImageJ and Imaris were 5845/6070 and 751,812/855,953 (μm^2^) (with proportions of 1:1.0385 and 1.1385), respectively (Fig. 1g, h, Additional file 7: Dataset S1). The average cell areas were 117.8473 and 126.3316 (μm^2^) (with a proportion of 1:1.0720), respectively (Fig. 1i). There were no obvious differences in the frequency distributions of cell areas based on these two results (Fig. 1j). Pearson correlation analysis also suggested that the cell areas were extremely similar based on comparisons of ImageJ/ImageJ, ImageJ/Imaris, and Imaris/Imaris results (Fig. 1k). Although Imaris has a friendly user interface and diverse statistical visualizations, some functions are not free of charge, and it can only export cell area values calculated based on the number of voxels. Consequently, we chose ImageJ and SR-Tesseler, two freely available open-source software packages, to develop an efficient procedure to characterize, segment, and quantify complex multicellularity in plants based on raw microscopy images.

### Overview of the procedure for quantifying and segmenting plant cells

**Fig. 2:**
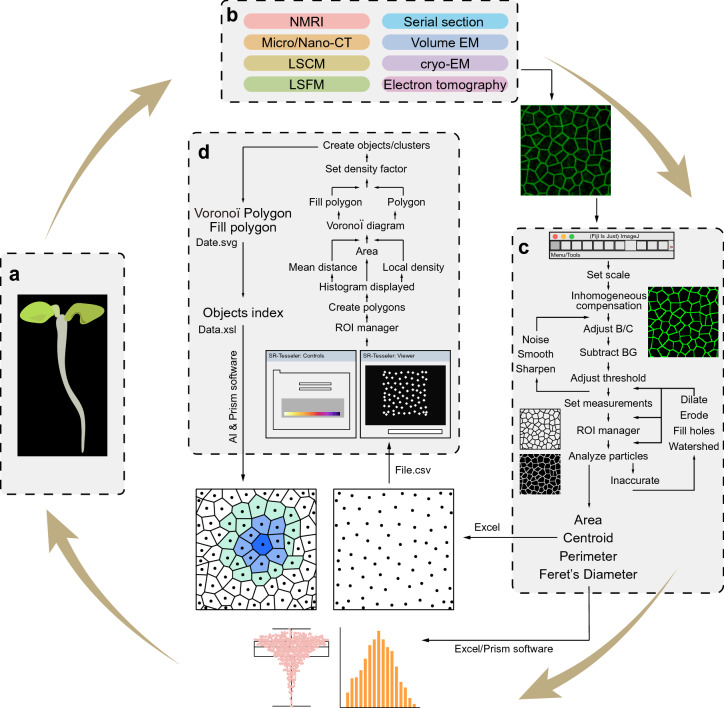
Flowchart of the procedure. **a** A plant sample (*Arabidopsis* seedling) prepared for analysis. **b** Basic raw imaging data for cellular outlines acquired by various 2D (two dimensional) and 3D imaging techniques used for this procedure; large-scale 3D images can be split into arbitrary 2D sections if needed. **c** Pre-processing, clarity adjustment, and parameter identification by ImageJ software. **d** Polygon creation, establishment of a Voronoï diagram, and object/cluster identification together with quantitative data generated and exported by SR-Tesseler software

Here, we describe how to recognize and quantify multicellular parameters from raw images using ImageJ and how to perform segmentation and organization analysis of plant cells based on their centroids with SR-Tesseler. The entire procedure, which is summarized in Fig. 2, consists of four modules: (i) plant tissue material preparation (Fig. 2a); (ii) collection of basic raw imaging data (acquired by various 2D and 3D imaging techniques) (Fig. 2b); (iii) image pre-processing and parameter identification (Fig. 2c); and (iv) centroid data conversion and generation of a Voronoï diagram (Fig. 2d).

**Fig. 3:**
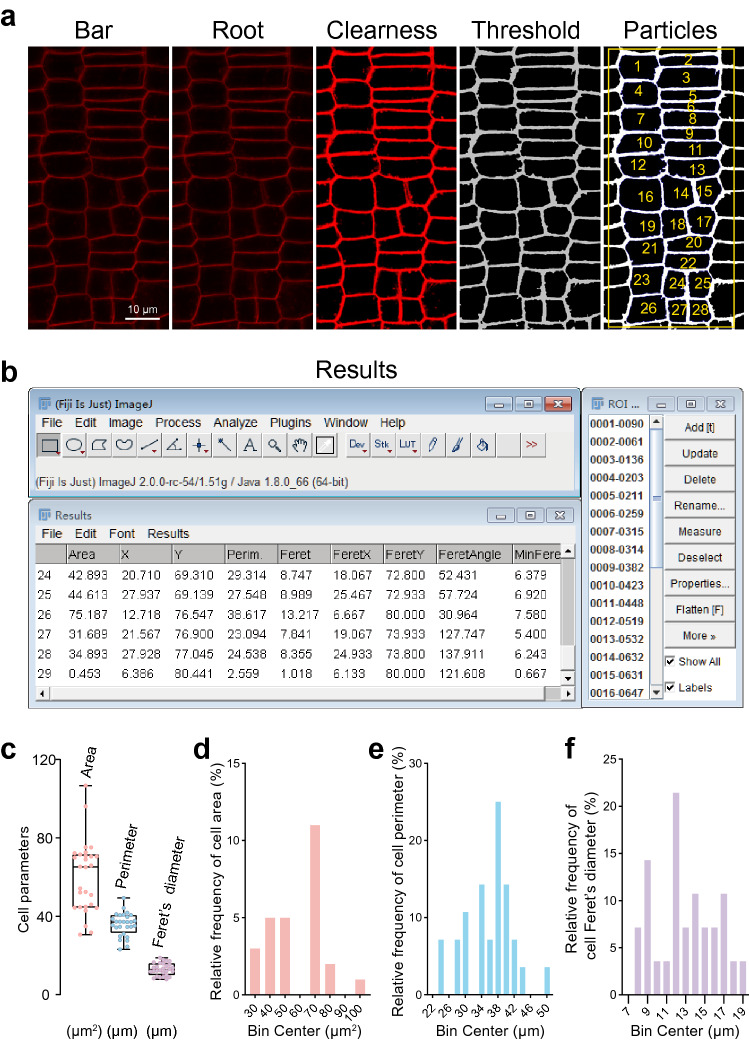
Recognition and qualification of cells in a vertical *Arabidopsis* root section. **a** Images of mCherry-labeled *Arabidopsis* root cells captured by Laser scanning confocal microscopy (LSCM) and images after clearness, threshold adjustment, and particle recognition, bar = 10 μm. **b** Graphical user interface, including the main interface, ROI (region of interest) manager panel, and the results display window of ImageJ software. The ROI list and results of the cellular parameters were identified and characterized, respectively, from *Arabidopsis* root cells in **a**. **c**–**f** Parameters of *Arabidopsis* root cells. Quantification of area, perimeter, and Feret’s diameter of *Arabidopsis* root cells in **a**. Boxplots represent mean, 25th, and 75th quartiles, whiskers represent minimum and maximum. n = 58 cells (**c**). Relative frequency distribution (percentage, %) analysis of area (**d**), perimeter (**e**), and Feret’s diameter (**f**) shown in **c**

To help users become familiar with the functionalities of this procedure, we provided several datasets of typical cell outline images and centroid data in .csv format (Figs. 3a, 5a, 7a, Additional file 4: Table S1, Additional file 9: Dataset S3, Additional file 12: Dataset S6 and Additional file 15: Dataset S9). Users can install and run ImageJ and SR-Tesseler on these datasets before using their own data. Finally, we included three raw microscopy images captured from three different plant tissues by LSCM, Micro CT, and semithin section light microscope imaging to demonstrate the practicality and reliability of our approach.

### Analysis of root cell images captured by LSCM

The first example shows an mCherry-labeled plasma membrane (PM) protein expressed in *Arabidopsis* root cells obtained by LSCM (Fig. 3a left). After performing clarity adjustment with ImageJ, the outlines of root cells were distinguishable and suitable for subsequent binary transform (Fig. 3a middle). We performed cell identification using the *Analyze Particles* plugin, and the results emerged in a new window with data information (Fig. 3b, Additional file 8: Dataset S2). Twenty-eight cells were successfully identified, while incomplete cells (split by the edges) were excluded from ROI (region of interest) selection (Fig. 3a right). All cell outlines can be found in the ROI manager windows on the right side of Fig. 3b. Boxplots were generated and relative frequencies of cell area, perimeter, and Feret’s diameter values were analyzed with GraphPad Prism software. The median values of the area, perimeter, and Feret’s diameter were 65.15 μm^2^, 37.07 μm, and 12.83 μm, respectively (Fig. 3c). All of these parameters presented an approximately Gaussian distribution after frequency distribution analysis (Fig. 3d–f).

**Fig. 4:**
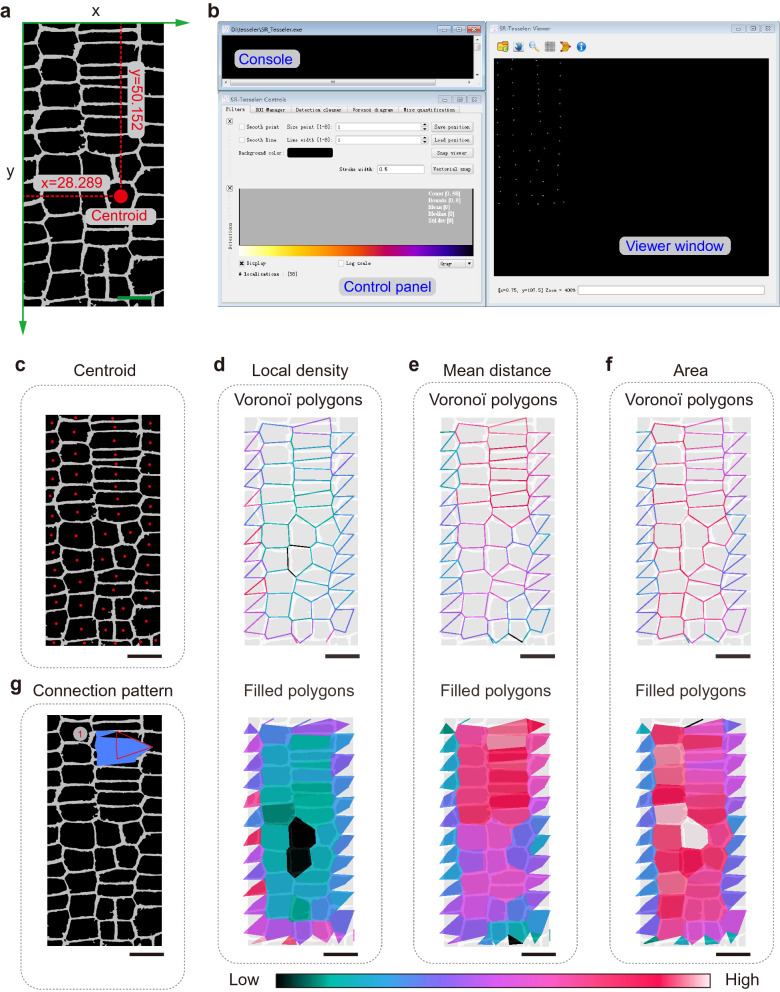
Analysis of *Arabidopsis* root cell organization by tessellation-based automatic segmentation of an LSCM image. **a** Illustration of a centroid coordinate read-out in *Arabidopsis* root cells generated by ImageJ. Green arrows indicate the direction of the x and y axes; the number indicates the centroid coordinate of the red point. **b** Graphical user interface of SR-Tesseler software, including the console, control window, and viewer panel. **c** Merged image of *Arabidopsis* root cell particles identified by ImageJ and centroid (red points) displayed in the SR-Tesseler viewer window. **d**–**f** Segmentation and quantification of experimental data according to the centroid shown in **c**. Polygon creation and establishment of Voronoï diagrams based on local density (**d**), mean distance (**e**), and area (**f**). Empty polygons are shown above, and filled polygons are shown below. All polygons were merged with the particles identified from *Arabidopsis* root cells. The polygons were pseudocolor-coded with respect to the segmentation results. **g** The connection pattern of clusters calculated from established objects. All the bars in this figure represent 10 μm

Next, we exported the centroid coordinates obtained from cell particle identification (Additional file 1: Fig. S1a and Additional file 9: Dataset S3); the location of a selected coordinate is shown in Fig. 4a. After converting the data into a .csv file, we performed multicellularity segmentation of the centroid data using SR-Tesseler software. After importing the modified centroid data into SR-Tesseler, three windows appeared, including a console for application messages, a control panel, and a data viewer that displayed the dots of the centroid (Fig. 4b). By merging this information with binarization of the raw image, the centroids of each cell were precisely located (Fig. 4c).

We then performed Voronoï tessellation by histogram adjustment or by modifying the density factor object creation. Following this step, the segmentation results were shown as pseudocolor-labeled polygons (Additional file 1: Fig. S1b–d). After merging the polygons with a modified raw image, the polygons exactly overlapped with the PM of each cell outline. The polygons could be also altered by setting thresholds for their average localization density, mean distance, and area (Fig. 4d–f). Furthermore, cell clusters could be created by changing the density factor cluster definition. Based on the mean distance threshold setting, we created cell clusters and found that they were located on the long, narrow cells in the top right corner of the image (Fig. 4g and Additional file 1: Fig. S1e). Finally, the Voronoï diagram and information about the cluster data were exported from the *Filters and Clusters* tab (Additional file 10: Dataset S4).

### Analysis of seed cell images captured by Micro-CT

**Fig. 5:**
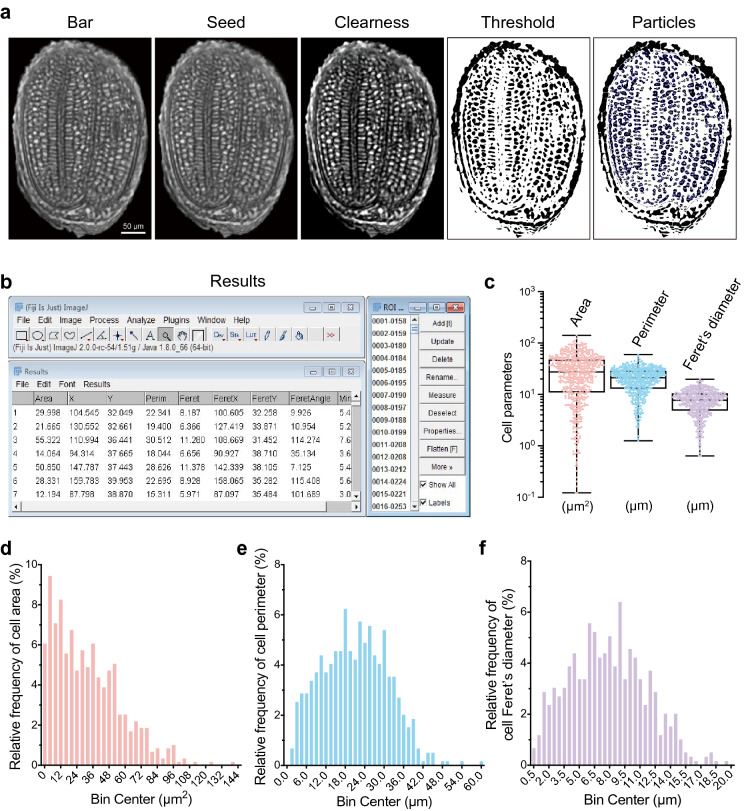
Recognition and qualification of cells in an *Arabidopsis* seed section. **a** Images of *Arabidopsis* seed cells captured by micro-computed tomography (Micro-CT) and images after clearness, threshold adjustment, and particle recognition, bar = 50 μm. **b** Graphical user interface of ImageJ software, including the main interface, ROI manager panel, and results display window. An ROI list and cellular parameters characterized from *Arabidopsis* seed cells in **a**. **c**–**f** Cellular parameters of *Arabidopsis* seed cells. Quantification of area, perimeter, and Feret’s diameter of *Arabidopsis* seed cells in **a**. Boxplots represent mean, 25th, and 75th quartiles, whiskers represent minimum and maximum, n = 593 cells (**c**). Relative frequency distribution (percentage, %) analysis of area (**d**), perimeter (**e**), and Feret’s diameter (**f**) shown in **c**

The second sample was an image of an *Arabidopsis* seed captured by Micro-CT (Fig. 5a). After scanning the whole seed, we performed 3D reconstruction. We choose a translation slice that clearly showed double cotyledons and a hypocotyl with cells inside (Fig. 5a). We then performed pre-processing and particle analysis and obtained all of the parameters from 593 cells in less than 15 min (Fig. 5b) (Additional file 11: Dataset S5). Boxplots showed that the median value of cell area, perimeter, and Feret’s diameter were 27.36 μm^2^, 21.08 μm, and 7.727 μm, respectively (Fig. 5c). The relative frequency of perimeter and Feret’s diameter values presented an approximately Gaussian distribution (Fig. 5d–f). Most of the cell areas ranged from 0 to 90 μm^2^, but a few were over 100 μm^2^ (Fig. 5d, Additional file 12: Dataset S6).

**Fig. 6:**
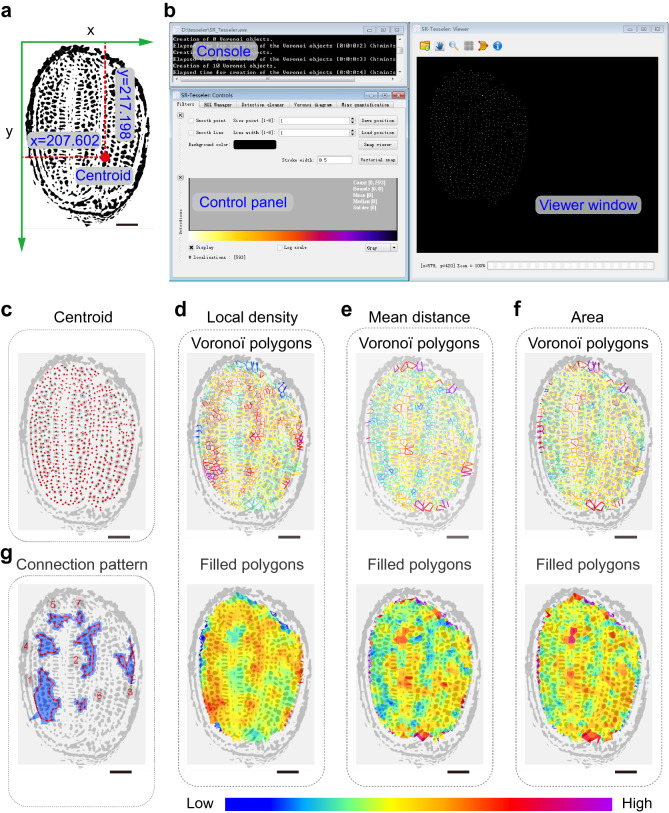
Analysis of *Arabidopsis* seed cell organization by tessellation-based automatic segmentation of a Micro-CT image. **a** Illustration of a centroid coordinate read-out in *Arabidopsis* seed cells produced by ImageJ. Green arrows indicate the direction of the x and y axes, and the number represents the centroid coordinate of the red point. **b** Graphical user interface of SR-Tesseler software, including the console, control window, and viewer panel. **c** Merged image of *Arabidopsis* seed cell particles identified by ImageJ and centroid (red points) displayed in the SR-Tesseler viewer window. **d**–**f** Segmentation and quantification of experimental data according to the centroid shown in **c**. Polygon creation and establishment of Voronoï diagrams based on local density (**d**), mean distance (**e**), and area (**f**). Empty polygons are shown above, and filled polygons are shown below. All polygons were merged with the particles identified from *Arabidopsis* seed cells. The polygons were pseudocolor-coded with respect to the segmentation results. **g** The connection patterns of clusters calculated from established objects. All bars in this figure represent 50 μm

We performed Voronoï tessellation using SR-Tesseler software (Fig. 6a, b). Excluding the seed coat, 593 centroids were precisely located in cells when merged with the raw image (Fig. 6c and Additional file 2: Fig. S2a). The Voronoï diagrams appeared to be slightly different after setting thresholds for these polygons at the average localization density, mean distance, and area (Fig. 6d–f, Additional file 2: Fig. S2b–d). Notably, we gained seven clusters after cluster creation by setting thresholds at the average localization density with a density factor of 1.15 in the *Clusters definition* option (Fig. 6g). The majority of these clusters were spread over the two cotyledons, suggesting that these cell clusters have a unique function resulting from their specific connection (Fig. 6g, Additional file 2: Fig. S2e, Additional file 13: Dataset S7).

### Analysis of a stem cell section captured by optical microscopy

**Fig. 7:**
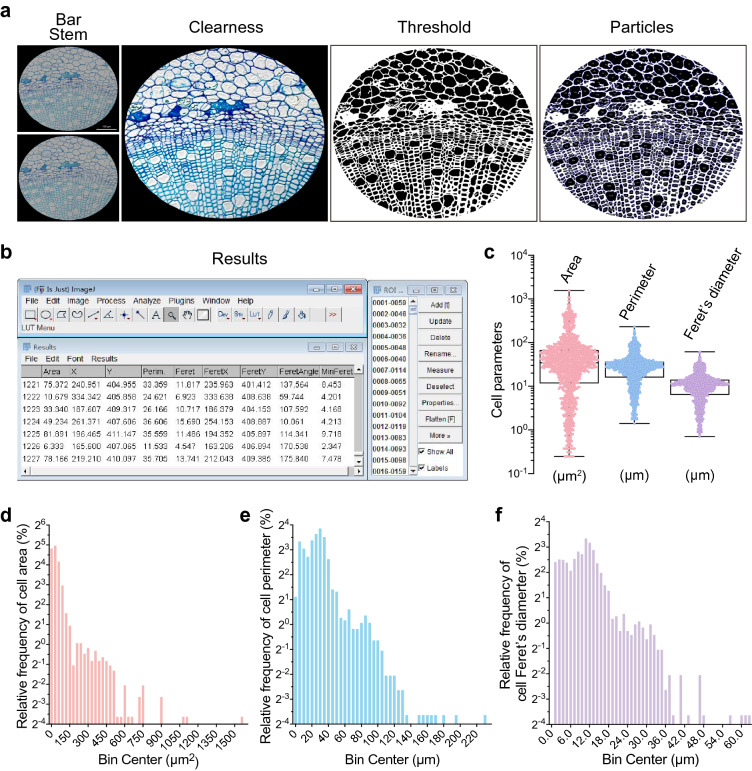
Recognition and qualification of cells in a transverse section of a *Populus trichocarpa* stem. **a** Images of *Populus trichocarpa* stem cells captured by light microscopy (LM) after staining with toluidine blue and images after clearness, threshold adjustment and particle recognition, bar = 100 μm. **b** Graphical user interface of ImageJ software, including the main interface, ROI manager panel, and results display window. ROI list and the cellular parameters characterized from *Populus trichocarpa* stem cells in **a**. **c**–**f** Cellular parameters of *Arabidopsis* seed cells. Quantification of area, perimeter, and Feret’s diameter of *Arabidopsis* seed cells in **a**. Boxplots represent mean, 25th, and 75th quartiles, whiskers represent minimum and maximum, n = 1243 cells (**c**). Relative frequency distribution (percentage, %) analysis of area (**d**), perimeter (**e**), and Feret’s diameter (**f**) shown in **c**

We also tested this procedure using an optical microscopy image of a transverse section of a *Populus trichocarpa* stem after staining the cells with toluidine blue (Fig. 7a). After pre-processing and particle analysis, we obtained the relevant parameters from 1243 cells in less than 20 min (Fig. 7b, Additional file 14: Dataset S8). Boxplots showed that the median value of area, perimeter, and Feret’s diameter were 34.46 μm^2^, 27.43 μm, and 10.79 μm, respectively (Fig. 7c). All cell parameters presented a non-Gaussian distribution following frequency distribution analysis (Fig. 7d–f). Interestingly, all of the distribution results showed three obvious peaks, indicating that at least three distinct cell types are present in *Populus trichocarpa* stems (Fig. 7d–f, Additional file 15: Dataset S9).

**Fig. 8:**
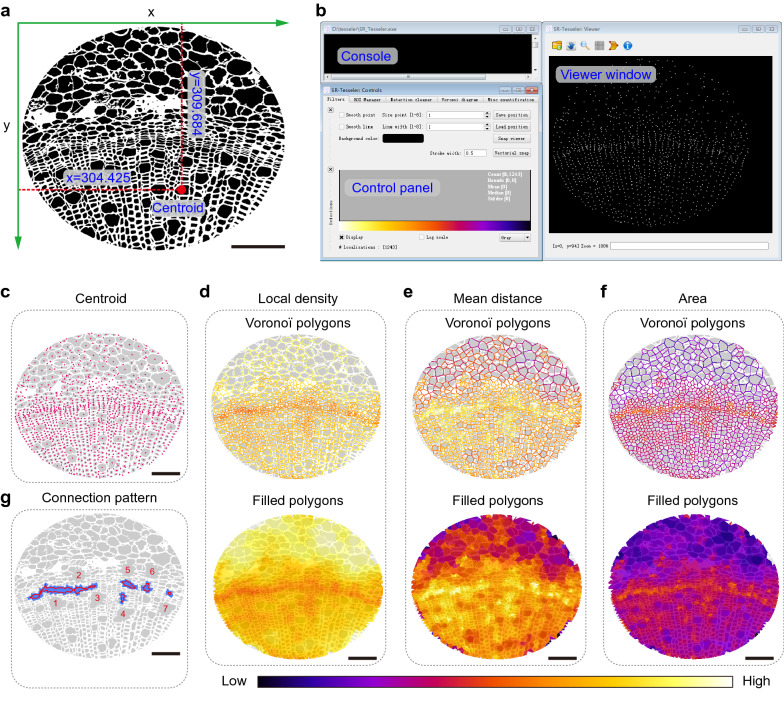
Analysis of *Populus trichocarpa* stem cell organization by tessellation-based automatic segmentation of an LM image. **a** Illustration of a centroid coordinate read-out in *Populus trichocarpa* stem cells produced by ImageJ. Green arrows indicate the direction of the x and y axes, and the number indicates the centroid coordinate of the red point. **b** Graphical user interface of SR-Tesseler software, including the console, control window, and viewer panel. **c** Merged image of *Populus trichocarpa* stem cell particles identified by ImageJ and centroid (red points) displayed in the SR-Tesseler viewer window. **d**–**f** Segmentation and quantification of experimental data according to the centroid in **c**. Polygon creation and establishment of Voronoï diagrams based on the local density (**d**), mean distance (**e**), and area (**f**). Empty polygons are shown above, and filled polygons are shown below. All polygons were merged with the particles identified from *Populus trichocarpa* stem cells. The polygons were pseudocolor-coded with respect to the segmentation results. **g** The connection pattern of clusters calculated from established objects. All bars in this figure represent 10 μm

We then performed Voronoï tessellation (Fig. 8a–g), finding that the 1243 centroids were also precisely located in cells when merged with the raw image (Fig. 8c, Additional file 3: Fig. S3a). Voronoï diagrams generated using three different threshold settings are shown in Fig. 8d–f (Additional file 3: Fig. S3b–d). Remarkably, we gained seven clusters after cluster creation by setting thresholds at the average area with a density factor of 2.3 in the *Clusters definition* option (Fig. 8g, Additional file 3: Fig. S3e, Additional file 16: Dataset S10). All of the clusters were distributed in cells of the vascular cambium.

## Discussion

Plant biology research targets complex multilevel systems with a high level of functional organization [1]. With the development of advanced imaging technology, high-resolution and large-scale images contain much data about cellular structure and a variety of cellular-based organic information [8–11]. Therefore, methods must be developed for high efficiency, high-quality, high-throughput identification and analysis of multicellular systems. MorphoGraphX is an open-source platform for the visualization and analysis of 4D biological datasets [34–37]. Although MorphoGraphX can be used for simple cell identification, this software package must be run on a computer with Linux Mint 19, Ubuntu 18.04 with Cuda 9.1, or Windows (64-bit) with Cuda 7.0 with 32–64 Gb of RAM and an adequate nVidia graphics card. Bitplane Imaris software generates a variety of valuable cellular parameters and clear visual displays by performing cell segmentation without compensating for non-homogeneous signals. Nevertheless, Imaris provides only cell areas calculated from a number of voxels, and its calculation functions are not free-of-charge.

A comparison of results obtained using ImageJ vs. Imaris revealed that both software packages produce precise cellular parameters, making ImageJ the software package of choice since it is freely available and highly effective. Using ImageJ, our newly developed procedure provided high-throughput cell recognition from raw images. The use of raw images with nonuniform section staining or inhomogeneous signals might result in indistinct cell outlines and reduce the precision of identification. To compensate for patchy signals, we suggest using the ‘Plane Brightness Adjustment’ plugin in ImageJ. In addition, manual ROI selection could be used to exclude incomplete cell data and improve the accuracy of data identification.

We identified cell centroids with ImageJ and further analyzed them using SR-Tesseler software. This analysis generated a rich set of data about intracellular connections and functional structures, providing a basis for identifying difficult-to-differentiate tissue structures. For instance, we can determine the location and direction of the vascular bundle in roots based on the pseudocolor graph by setting the threshold at localization density when clusters are created. In addition, the level of plant cell communication can be illustrated by the readout of pseudocolor images using the mean distance threshold setup. The distribution of different cell types can also be shown by setting the threshold at area; for example, using this technique, leaf epidermis can be distinguished from stomatal cells and root epidermal cells can be distinguished from cortical cells.

New recognition techniques for 3D multicellular images and the exploitation of novel Voronoï segmentation techniques will greatly facilitate the study of cell structure and function. The continued improvement of imaging approaches and identification techniques based on artificial intelligence should allow cells to be correctly identified more easily in the future.

## Conclusion

The procedure developed in this study allows users to perform recognition and qualification of multicellular micrographs with ImageJ (see “Methods”: Steps 1A(i–vii) and Steps 1B(i–iii)). Almost all types of raw images, such as fluorescently labeled cell wall/plasma membrane, stained sections, and slices from 3D reconstructions, can be processed during the first step of this procedure. Cell particle identification offers various cellular messages, providing sufficient information for further investigating plant tissue structure and dynamics. The centroids of each cell exported from ImageJ are then used to segment and calculate the connectivity and organization of plant cells based on Voronoï tessellation (see “Methods”: Steps 2A(i–iii), 2B(i–ix), and 2C(i–iii)). Collectively, this procedure can be used for reliable, high-efficiency cell identification and the evaluation of cell connectivity, facilitating the study of many developmental processes, biological homeostasis, and plant morphogenesis.

## Methods

### Required data files

Raw images of cellular outlines acquired from two-dimensional (2D) sections and 3D imaging techniques can be analyzed with this procedure. The format of the input images should be supported by ImageJ software, including tiff, png, gif, jpeg, bmp, gicom, fits files, and the like. Some images produced by various optical microscopy techniques, such as light sheet fluorescence microscopy (LSFM), laser scanning confocal microscopy (LSCM), and so on can be exported to a format supported by ImageJ using software provided with the microscope. Multi-scale 3D images generated by volume electron microscopy, LSFM, or micro/nano computed tomography (Micro/Nano CT) and so on should be split into arbitrary 2D sections with clear cellular outlines from the ROI after reconstruction and prior to analysis.

### Computer equipment

ImageJ, GraphPad Prism, and Adobe Illustrator software can be run on Windows XP, Vista, 7, 8, or 10, Mac OS X 10.8 “Mountain Lion” or later, or Linux with amd64 and ×86 architecture. Sufficient amounts of random-access memory (RAM) must be allocated to ImageJ/Java in order for all the images to be loaded. SR-Tesseler software can be run on Windows XP or Vista, 7, 8, or 10 based on 32- and 64-bit operating systems. Bitplane Imaris can be run on MS Windows 10 × 64 or Mac OS X 10.9 or later. A fully compatible OpenGL graphics card with as much memory as possible should be used. Sufficient RAM is required to store the images (8 GB is the absolute minimum).

### Software

- ImageJ (https://imagej.net/FIJI/Downloads).
- Plane Brightness Adjustment Plugin (https://imagej.nih.gov/ij/plugins/plane-brightness/index.htm).
- Java v.8 or later (https://www.java.com/en/download/).
- SR-Tesseler (http://www.iins.u-bordeaux.fr/team-sibarita-SR-Tesseler).
- Bitplane Imaris (https://imaris.oxinst.com/) (Demo edition).
- GraphPad Prism (https://www.graphpad.com/scientific-software/prism/).
- Adobe Illustrator (AI) (https://www.adobe.com/cn/products/illustrator/free-trial-download.html).

### Installation of ImageJ

To install ImageJ, follow the instructions below:

1. Download and install ImageJ (64-bit version) from https://imagej.net/FIJI/downloads (required java v.8 or later).
2. Choose Help > Update. Click *Show Details* to follow the progress and see more details.
3. Download the Plugins from https://imagej.nih.gov/ij/plugins/index.html and copy to the plugins folder under the root directory when needed.
4. Restart ImageJ.

### Plant materials

The fluorescent images of the *Populus trichocarpa* embryo section were acquired by LSFM (Lightsheet Z.1 Microscope System, Carl Zeiss, Germany) fitted with a 10× water-immersion imaging objective and two 5× illumination objectives. An optical section from an *Arabidopsis* seed was selected from thousands of slices in the 3D reconstruction results from a whole seed captured by Micro-CT (Skyscan 1173, Belgium). A fluorescent image of plasma membranes labeled with the fluorescent protein mCherry in *Arabidopsis* root cells was obtained by LSCM (TSC SP8, Leica, Germany). A 2 μm transverse section of a *Populus trichocarpa* stem was cut with a microtome (Leica-RM2265, Germany), stained with Toluidine Blue O (Sigma, USA), and imaged with a Leica Aperio VERSA digital pathology scanner (Leica, Germany).

### Cell identification and quantitation with Imaris

Imaris software was used to evaluate the veracity of cell identification by ImageJ. The following procedure was used: Import the image from *Imaris File Converter* or directly drop it into Imaris software (in a supported format). The image is then displayed in the Surpass view. Click on the *Add new Cells* icon to highlight the cell creation. Choose the last detection types of cells and click on the *Next* button. Two different detection algorithms can be used, depending on the cell staining technique and sample preparation (cytoplasm or cell membrane boundary). Click the *Cell Membrane Detection* button and choose the correct source channel of the raw image. Use two consecutive clicks to measure the diameter of the smallest cell and membrane detail before inputting the relevant measuring data and then click the *Next* button. After adjusting the cell membrane threshold based on intensity and quality, perform cell classification using various types of filters and then click the *Finish* button. Use the *Color* icon to define the pseudocolor and the *Statistics* icon to obtain the detailed cell parameter values.

### Compensation for non-homogeneous fluorescent signals

To overcome the problem of light attenuation in images acquired by fluorescence microscopy, non-homogeneous fluorescent signal distribution is compensated for using Plane Brightness Adjustment, a plugin in the ImageJ software package [38]. Download ‘Plane_Brightness_Adjustment.jar’ from https://imagej.nih.gov/ij/plugins/plane-brightness/index.htm and drop the plugin into the plugins > Stack folder. After clicking the Help > Refresh Menus command, the plugin can be found in the Plugins > Stacks menu item. 8-bit grayscale and RGB images can be processed using this plugin, which can also be used with image stacks. *Slope* indicates the maximum allowed change (an 8-bit integer) of the Lipschitz filter between the intensities of two neighboring pixels. *Threshold* indicates the pixel value at which the adjustment begins to work. The values below the threshold are not corrected. *Maxfactor* is a limit imposed on the factor by which the grayscale values are multiplied to increase brightness. Press *OK* to verify the application.

### Detailed steps of the procedure

1. Follow option A to identify the characteristics of a multicellular image; follow option B to analyze the cellular parameters and create figures.
  A. Recognition and qualification of a multicellular image. Total time: 21–32 min, including 1–2 min for Step 1A(i–ii); 10–15 min for Step 1A(iii–v); and 10–15 min for Step 1A(vi–vii).
    i. *Image import.* Start the ImageJ software. Import two images, including one with and one without a scale bar (Directly drag the images into ImageJ or click *File *>* Open *> to open the original images).
    ii. *Define the scale*. Select the *Straight* button from the ImageJ panel and draw a straight line the same length as the bar on the image. Click the *Analyze* >* Set scale* and type the true bar distance in the *known distance* text box and the unit in the *Unit of length* text box. Check *Global* to ensure that the same scale is applied to the other image (without the bar). Press the *OK* button to confirm and close the *Set scale* window. Close the image (with the bar) and retain the image (without the bar), which can be further analyzed (**Attention**).
    iii. *Clarity adjustment*. Click *Image* >* Adjust* >* Brightness/Contrast* to open the B&C window to adjust the brightness and contrast. Slide the slide block (from right to left) above *maximum* to improve the image brightness, and slide the slide block (from left to right) above the *minimum* to remove the background. Press *apply* for confirmation (**Attention**).
    iv. *Binary conversion* (*Threshold adjust*). Click *Image* >* Type *>* 32*-*bit* to convert the image to a bitmap file. Click *Image *> *Adjust *>* Threshold*, unchecking the *Dark background* text box to turn the intracellular signal red. Slide the slide block until the red areas emerge clearly and cover all the intracellular areas (Do not add any non-specific signal). Finally, press the *Apply* and *OK* buttons without checking the *set background pixels to NaN*. Black represents cellular outlines and white represents the intracellular regions (**Attention/Troubleshooting**).
    v. *Optimization of cellular outlines*. To better distinguish and identify the cell outlines, use the *Erode*, *Dilate*, *Fill holes*, and *Watershed* commands. In *Process* > *Binary*, *Erode* can be used to obtain better connections for cell outlines and *Dilate* can be used to enlarge intracellular signals. *Process *>* Binary* > *Fill holes* or *watershed* can be used to fill holes inside the cells or automatically divide a cell that should not be a single cell (**Attention**).
    vi. *Parametric recognition*. Click *Analyze *>* Set measurements* and check the parameters (*Area*, *Centroid*, *Perimeter*, *Feret’s diameter*, and more parameters if required) to determine what will be displayed in the results list. Click *Analyze *>* Analyze particles* and check *Display results*, *Exclude on edges*, and *Add to Manager*. Press *OK* to obtain the results. Finally, click *Edit *>* Invert* to change the intracellular parts to black to make the cells easier to recognize (**Attention/Troubleshooting**).
    vii. *Exceptional data exclusion and data export*. Determine the range of cell size based on the results list obtained in Step 1A(vi) and exclude cells with unusual sizes. Repeat Step 1A(vi) and correct the size (unit^2^) from the maximum to minimum value in the *Analyze Particles* window. If exceptional cells are still present in the results list, delete the particle in the *ROI Manager* manually and perform *More *> *Multi*-*Measure* on the *ROI Manager* window to show the corrected results list. Finally, export the data to *Excel* from the *Results* window (**Attention**).
  B. Cellular parameter analysis and figure creation. Total time: 20–30 min, including 5–10 min for Step 1B(i–ii) and 15–20 min for Step 1B(iii)
    i. *Data import.* Start GraphPad Prism (click the *Prism* icon) and choose the data table and options in the *Welcome to GraphPad Prism* window. Import the data that were exported during Step 1A(vii).
    ii. *Data analysis*. In *the Analysis toolbar* above, click the *analysis* button to open the *Analyze Data* window. Choose the analysis method from the list on the left and the data sets from the list on the right (for examples: choose *Column analyses* > *t*-*tests*/*ANOVA* to compare the differences among data sets, and choose *Column analyses* > *Frequency distribution* to generate a Frequency distribution histogram of the data).
    iii. *Figure creation*. Click *Graphs* > *Data 1* (can be renamed when needed) in the toolbar on the left and define a graph type in the *Change Graph Type* window. Modify the graph in the *Format Graph*/*Axes* window by double-clicking the graph. In the *Export toolbar* above, click the *export* icon to export the figure (**Attention**).
2. Follow option A to convert centroid data to .cvs format, which is supported by SR-Tesseler; follow option B to precisely and automatically quantify the cellular organization by presenting a segmentation framework based on Voronoï tessellation; follow option C to analyze the data and create a figure based on the Voronoï diagram images and objects/clusters data.
  A. Conversion of centroid data for SR-Tesseler software. Total time: 2–5 min for Step 1A(i–ii)
    i. *Material requested*. Open the data exported from 1A(vii) and create a new *Excel* file.
    ii. *Convert format*. Copy the x and y coordinates of the centroid recognized from the original image in Step 1A(vii). Paste the data to a newly created *Excel* file following the format in Additional file 4: Table S1. Export the data by saving the new data in .csv format. The first horizontal line should be *x[pix]*, *y[pix]*, *intensity*, and *frame*, respectively, and the x[pix], y[pix] column should be the x and y coordinates of the centroid. All of the values in the *intensity* column should be ‘0’, and all of the values in the *frame* column could be ‘1’ (**Attention**).
  B. Segmentation and organization analysis of plant cells. Total time: 10–20 min, including 2–5 min for Step 1A(i–v); 5–10 min for Step 1A(vi–vii); and 3–5 min for Step 1A(viii–ix).
    i. Launch *SR*-*Tesseler.exe* to start the program. Two windows will appear: a console for application messages and a data viewer. The GUI is shown in Figs. 4b, 6b and 8b.
    ii. *Data import*. Click the *open* button and select the localization data file exported from Step 2A(ii) (**Troubleshooting**).
    iii. Once the data are loaded, the cell coordinates are displayed in the *SR*-*Tesseler* viewer. Meanwhile, a control window with a histogram and options are displayed.
    iv. Under the first tab (*filters*), users can coordinate the background and dot color by clicking on the *background color* or select the color from the drop-down list in the lower right-hand corner (**Attention**).
    v. Under the *ROI Manager* tab, ROIs can be used during object and cluster creation. Click on the *polygon* icon to add an ROI, left click to add a new point to the current ROI, and double left click to complete the current ROI, which is automatically added to the ROI list.
    vi. *Create a Voronoï diagram*. Under the *Voronoï diagram* tab, a VoronoÏ diagram can be generated based on the initial detection dataset by clicking the *Create polygon* button; a histogram of *Local Densities* is displayed. A histogram of *Mean Distance* and *Area* can also be acquired by choosing the drop-down list under the histogram diagram. Check the *Fill polygon* checkbox to fill in the Voronoï polygons based on multicellular segment patterns. The cell boundaries are accurately displayed as polygons based on cell centroid dots. The polygons can be displayed with pseudocolors calculated based on the parameters local density, mean distance, and area, if required (**Attention**).
    vii. *Creating of objects/clusters*. Click the *Objects* tab under the *Voronoï diagram* tab for object creation. Here users can determine the patterns of different tightly connected cells displayed in *SR*-*Tesseler Viewer* by modifying the density factor and the min/max area (in pixel^2^)/localizations. After clicking *Set density factor* and the *Create objects* button, the object information (including *Obj index*, *Area*, *Detections*, *Circularity*, and *Diameter of objects*) is listed in the lower part of the *Objects* tab. The *Clusters* tab under the *Voronoï diagram* tab can be clicked to create clusters. (**Troubleshooting**).
    viii. *Data export*. After creating objects or clusters, the data can be exported by clicking the *Export stats* button under the *Objects* or *Clusters* tab (**Attention**).
    ix. *Graph export*. All graphs in the SR-Tesseler viewer window can be outputted in svg format under the *Filters* tab by clicking the *Voronoï snap* button. A merged image of the original figure and the Voronoï diagram can be created using *AI* software (**Attention**).
  C. Data analysis and figure creation. Total time: 17–24 min, including 1–2 min for Step 2C(i); 15–20 min for Step 2C(ii); and 1–2 min for Step 2C(iii)
    i. *Import data and data analysis*. The parameters objects and cluster export from 2B(viii) (*Obj index*, *Area*, *Detections*, *Circularity*, and *Diameter of objects*) can also be analyzed with GraphPad Prism by following Step 1B(ii).
    ii. *Merge the original figure and the Voronoï diagram*. Launch *AI* software and create a new artboard in *A4* size with the *RGB* system. Directly pull the images or click *File *>* Open* to open the original image and the Voronoï diagram image exported from 2B(ix). Remove the background of the Voronoï diagram image and copy-paste to the same board of the original image. Adjust the size and align the two images until they are exactly lined up (**Attention/Troubleshooting**).
    iii. *Export the merged image*. Click the *Document setup* button above the toolbar and click *artboard tools* for board adjustment. In the *File* list, click *Export *>* Export as* to export the image. Add the name of the image and choose the format before clicking the *export* button.
3. All **Attention** and **Troubleshooting** information is available in Additional file 5: Table S2, Additional file 6: Table S3. The movie of the actual operating procedure is available in Additional file 17: Movie S1.

## Supplementary information

**Additional file 1: Figure S1.** Centroids, polygon creation, establishment of a Voronoï diagram, and object/cluster identification in *Arabidopsis* root cells.

**Additional file 2: Figure S2.** Centroids, polygon creation, establishment of a Voronoï diagram, and object/cluster identification of cells in an *Arabidopsis* seed section.

**Additional file 3: Figure S3.** Centroids, polygon creation, establishment of a Voronoï diagram, and object/cluster identification of cells in a transverse section of a *Populus trichocarpa* stem.

**Additional file 4: Table S1.** Example data from SR-Tesseler software.

**Additional file 5: Table S2.** Attention items in this procedure.

**Additional file 6: Table S3.** Troubleshooting in this procedure.

**Additional file 7: Dataset S1.** Parameter identification of *Populus trichocarpa* embryo cells by ImageJ and Imaris software.

**Additional file 8: Dataset S2.** Parameter identification of mCherry-labeled cells in a vertical *Arabidopsis* root section by ImageJ software.

**Additional file 9: Dataset S3.** Modification of centroid coordinates of cells in a vertical *Arabidopsis* root section for analysis with SR-Tesseler software.

**Additional file 10: Dataset S4.** The objects stats of cells in a vertical *Arabidopsis* root section using SR-Tesseler software.

**Additional file 11: Dataset S5.** Parameter identification of cells in an *Arabidopsis* seed section by ImageJ software.

**Additional file 12: Dataset S6.** Modification of centroid coordinates of cells in an *Arabidopsis* seed section for analysis with SR-Tesseler software.

**Additional file 13: Dataset S7.** The objects stats of cells in an *Arabidopsis* seed section using SR-Tesseler software.

**Additional file 14: Dataset S8.** Parameter identification of toluidine blue-labeled cells in a transverse section of a *Populus trichocarpa* stem by ImageJ software.

**Additional file 15: Dataset S9.** Modification of centroid coordinates of cells in a transverse section of a *Populus trichocarpa* stem for analysis with SR-Tesseler software.

**Additional file 16: Dataset S10.** The objects stats of cells in a transverse section of a *Populus trichocarpa* stem by SR-Tesseler software.

**Additional file 17: Movie S1.** Movie of the actual operating procedure.

## Data Availability

All data generated or analyzed during this study are included in this published article and Additional files 1, 2, 3, 4, 5, 6, 7, 8, 9, 10, 11, 12, 13, 14, 15, 16 and 17.

## References

[1] Knoll AH (2011). The multiple origins of complex multicellularity. Annu Rev Earth Plant Sci.

[2] Cui YN, Zhang X, Yu M, Zhu YF, Xing JJ, Lin JX (2019). Techniques for detecting protein-protein interactions in living cells: principles, limitations, and recent progress. Sci China Life Sci.

[3] Wang L, Xue YQ, Xing JJ, Song K, Lin JX (2018). Exploring the spatiotemporal organization of membrane proteins in living plant cells. Annu Rev Plant Biol.

[4] Zhang X, Cui YN, Yu M, Lin JX (2019). Single-molecule techniques for imaging exo-endocytosis coupling in cells. Trends Plant Sci.

[5] Wang XH, Li XJ, Deng X, Luu DT, Maurel C, Lin JX (2015). Single-molecule fluorescence imaging to quantify membrane protein dynamics and oligomerization in living plant cells. Nat Protoc.

[6] Benitez-Alfonso Y, Faulkner C, Pendle A, Miyashima S, Helariutta Y, Maule A (2013). Symplastic intercellular connectivity regulates lateral root patterning. Dev Cell.

[7] Kaiser D (2001). Building a multicellular organism. Annu Rev Genet.

[8] Ovecka M, von Wangenheim D, Tomancak P, Samajova O, Komis G, Samaj J (2018). Multiscale imaging of plant development by light-sheet fluorescence microscopy. Nat Plants.

[9] Titze B, Genoud C (2016). Volume scanning electron microscopy for imaging biological ultrastructure. Biol Cell.

[10] Clark NM, Van den Broeck L, Guichard M, Stager A, Tanner HG, Blilou L (2020). Novel imaging modalities shedding light on plant biology: start small and grow big. Annu Rev Plant Biol..

[11] Shen WW, Ma LY, Zhang X, Li XX, Zhao YY, Jing YP (2020). Three-dimensional reconstruction of *Picea wilsonii* Mast. pollen grains using automated electron microscopy. Sci China Life Sci.

[12] Olle-Vila A, Duran-Nebreda S, Conde-Pueyo N, Montanez R, Sole R (2016). A morphospace for synthetic organs and organoids: the possible and the actual. Integr Biol.

[13] Schindelin J, Rueden CT, Hiner MC, Eliceiri KW (2015). The ImageJ ecosystem: an open platform for biomedical image analysis. Mol Reprod Dev.

[14] Arena ET, Rueden CT, Hiner MC, Wang S, Yuan M, Eliceiri KW (2017). Quantitating the cell: turning images into numbers with ImageJ. WIERs Dev Biol.

[15] Polan DF, Brady SL, Kaufman RA (2016). Tissue segmentation of computed tomography images using a Random Forest algorithm: a feasibility study. Phys Med Biol.

[16] Cardona A, Saalfeld S, Schindelin J, Arganda-Carreras I, Preibisch S, Longair M (2012). TrakEM2 software for neural circuit reconstruction. PLoS ONE.

[17] Zhang SL, Wu GJ, Yang XG, Jiang WH, Zhou JW (2018). Digital image-based identification method for the determination of the particle size distribution of dam granular material. KSCE J Civ Eng.

[18] Cui YN, Li XJ, Yu M, Li RL, Fan LS, Zhu YF (2018). Sterols regulate endocytic pathways during flg22-induced defense responses in *Arabidopsis*. Development.

[19] Xing JJ, Li XJ, Wang XH, Lv XQ, Wang L, Zhang L (2019). Secretion of phospholipase Dδ delta functions as a regulatory mechanism in plant innate immunity. Plant Cell.

[20] Wang L, Li H, Lv XQ, Chen T, Li RL, Xue YQ (2015). Spatiotemporal dynamics of the BRI1 receptor and its regulation by membrane microdomains in living *Arabidopsis* cells. Mol Plant.

[21] Zhang X, Cui Y, Yu M, Su B, Gong W, Baluska F (2019). Phosphorylation-mediated dynamics of nitrate transceptor NRT1.1 regulate auxin flux and nitrate signaling in lateral root growth. Plant Physiol.

[22] Peter FA, Ethan DB (1986). Generalized dirichlet tessellations. Geom Dedicata.

[23] Maurer CR, Qi R, Raghavan V (2003). A linear time algorithm for computing exact euclidean distance transforms of binary images in arbitrary dimensions. IEEE T Pattern Anal.

[24] Brostow W, Dussault JP, Fox BL (1978). Construction of Voronoi polyhedra. J Comp Phys.

[25] Skamarock WC, Klemp JB, Duda MG, Fowler LD, Park SH, Ringler TD (2012). A multiscale nonhydrostatic atmospheric model using centroidal voronoi tesselations and C-Grid staggering. Mon Weather Rev.

[26] Du Q, Gunzburger M, Ju LL (2010). Advances in studies and applications of centroidal Voronoi Tessellations. Numer Math Theory Methods Appl.

[27] Passolt G, Fix MJ, Toth SF (2013). A Voronoi tessellation based approach to generate hypothetical forest landscapes. Can J For Res.

[28] Hu H, Liu XH, Hu P (2014). Voronoi diagram generation on the ellipsoidal earth. Comput Geosci.

[29] Bock M, Tyagi AK, Kreft JU, Alt W (2010). Generalized Voronoi Tessellation as a model of two-dimensional cell tissue dynamics. Bull Math Biol.

[30] Levet F, Hosy E, Kechkar A, Butler C, Beghin A, Choquet D (2015). SR-Tesseler: a method to segment and quantify localization-based super-resolution microscopy data. Nat Methods.

[31] Perraki A, Gronnier J, Gouguet P, Boudsocq M, Deroubaix AF, Simon V (2018). REM1.3′s phospho-status defines its plasma membrane nanodomain organization and activity in restricting PVX cell-to-cell movement. PLoS Pathog.

[32] Gronnier J, Crowet JM, Habenstein B, Nasir MN, Bayle V, Hosy E (2017). Structural basis for plant plasma membrane protein dynamics and organization into functional nanodomains. Elife.

[33] Nicovich PR, Owen DM, Gaus K (2017). Turning single-molecule localization microscopy into a quantitative bioanalytical tool. Nat Protoc.

[34] Barbier de Reuille P, Routier-Kierzkowska AL, Kierzkowski D, Bassel GW, Schupbach T, Tauriello G (2015). MorphoGraphX: a platform for quantifying morphogenesis in 4D. Elife.

[35] Sapala A, Runions A, Routier-Kierzkowska AL, Das Gupta M, Hong L, Hofhuis H (2018). Why plants make puzzle cells, and how their shape emerges. Elife.

[36] Jackson MDB, Duran-Nebreda S, Kierzkowski D, Strauss S, Xu H, Landrein B (2019). Global topological order emerges through local mechanical control of cell divisions in the *Arabidopsis* shoot apical meristem. Cell Syst.

[37] Montenegro-Johnson TD, Stamm P, Strauss S, Topham AT, Tsagris M, Wood ATA (2015). Digital single-cell analysis of plant organ development using 3DCellAtlas. Plant Cell.

[38] Michálek J, Čapek M, Kubínová L (2010). Compensation of inhomogeneous fluorescence signal distribution in 2D images acquired by confocal microscopy.. Microsc Res Techniq..

