# Supplementary material for: High-efficiency procedure to characterize, segment, and quantify complex multicellularity in raw micrographs in plants
Source: Plant Methods. 2020 Jul 28;16:100. doi: 10.1186/s13007-020-00642-0 (PMC7390866; doi:10.1186/s13007-020-00642-0)
Supplement: Supplementary file 1 — Additional file 1: Figure S1. Centroids, polygon creation, establishment of a Voronoï diagram, and object/cluster identification in Arabidopsis root cells. [file 13007_2020_642_MOESM1_ESM.docx]

**Supplementary information**

**High-efficiency procedure to characterize, segment, and quantify complex multicellularity in raw micrographs in plants**

Xi Zhang^2,3^, Zijian Hu^2,3^, Yayu Guo^2^, Xiaoyi Shan^2^, Xiaojuan Li^1,2^, Jinxing Lin^1,2^*

^1^Beijing Advanced Innovation Center for Tree Breeding by Molecular Design, Beijing Forestry University, Beijing 10083, China

^2^College of Biological Sciences and Biotechnology, Beijing Forestry University, Beijing 10083, China

^3^These authors contributed equally: Xi Zhang, Zijian Hu

*e-mail: [linjx@ibcas.ac.cn](mailto:linjx@ibcas.ac.cn)

***This pdf file includes:***

**Additional file 1-3: Figures S1-3**

**Figure S1.** Centroids, polygon creation, establishment of a Voronoï diagram, and object/cluster identification in *Arabidopsis* root cells.

**Figure S2.** Centroids, polygon creation, establishment of a Voronoï diagram, and object/cluster identification of cells in an *Arabidopsis* seed section.

**Figure S3.** Centroids, polygon creation, establishment of a Voronoï diagram, and object/cluster identification of cells in a transverse section of a *Populus trichocarpa* stem.

**Additional file 4-6: Tables S1-3**

**Table S1.** Example data for SR-Tesseler software.

**Table S2.** Attention items in this procedure.

**Table S3.** Troubleshooting in this procedure.

Additional file 1: Figure S1

**Additional file 1: Figure S1** Centroids, polygon creation, establishment of a Voronoï diagram, and object/cluster identification in *Arabidopsis* root cells. **a** Image of the centroid (red points) of *Arabidopsis* root cell particles identified by ImageJ. **b-d** Segmentation and quantification of experimental data according to the centroid shown in **a**. Original images of polygon creation and establishment of Voronoï diagrams based on local density (**b**), mean distance (**c**), and area (**d**). Empty polygons are shown above, and filled polygons are shown below. All polygons were merged with the particles identified from *Arabidopsis* root cells. The polygons were pseudocolor-coded with respect to the segmentation results. **e** The connection pattern of clusters calculated from established objects. All the bars in this figure represent 10 μm.
