# Supplementary material for: High-efficiency procedure to characterize, segment, and quantify complex multicellularity in raw micrographs in plants
Source: Plant Methods. 2020 Jul 28;16:100. doi: 10.1186/s13007-020-00642-0 (PMC7390866; doi:10.1186/s13007-020-00642-0)
Supplement: Supplementary file 3 — Additional file 3: Figure S3. Centroids, polygon creation, establishment of a Voronoï diagram, and object/cluster identification of cells in a transverse section of a Populus trichocarpa stem. [file 13007_2020_642_MOESM3_ESM.docx]

Additional file 3: Figure S3

**Additional file 3: Figure S3** Centroids, polygon creation, establishment of a Voronoï diagram, and object/cluster identification of cells in a transverse section of a *Populus trichocarpa* stem. **a** Image of the centroid (red points) of *Populus trichocarpa* stem cell particles identified by ImageJ. **b-d** Segmentation and quantification of experimental data according to the centroid shown in **a**. Original images of polygon creation and establishment of Voronoï diagrams based on local density (**b**), mean distance (**c**), and area (**d**). Empty polygons are shown above, and filled polygons are shown below. All polygons were merged with the particles identified from *Populus trichocarpa* stem cells. The polygons were pseudocolor-coded with respect to the segmentation results. **e** The connection pattern of clusters calculated from established objects, All the bars in this figure represent 100 μm.
