# Supplementary material for: High-efficiency procedure to characterize, segment, and quantify complex multicellularity in raw micrographs in plants
Source: Plant Methods. 2020 Jul 28;16:100. doi: 10.1186/s13007-020-00642-0 (PMC7390866; doi:10.1186/s13007-020-00642-0)
Supplement: Supplementary file 4 — Additional file 4: Table S1. Example data from SR-Tesseler software. [file 13007_2020_642_MOESM4_ESM.docx]

**Additional file 4: Table S1** Example data for SR-Tesseler software

| **Example data for SR-Tesseler software** | | | |
| --- | --- | --- | --- |
| **x[pix]** | **y[pix]** | **intensity** | **frame** |
| x 1 | y 1 | 0 | 1 |
| x 2 | y 2 | 0 | 1 |
| x 3 | y 3 | 0 | 1 |
| … | … | 0 | 1 |
| x n-1 | y n-1 | 0 | 1 |
| x n | y n | 0 | 1 |
