# Supplementary material for: High-efficiency procedure to characterize, segment, and quantify complex multicellularity in raw micrographs in plants
Source: Plant Methods. 2020 Jul 28;16:100. doi: 10.1186/s13007-020-00642-0 (PMC7390866; doi:10.1186/s13007-020-00642-0)
Supplement: Supplementary file 5 — Additional file 5: Table S2. Attention items in this procedure. [file 13007_2020_642_MOESM5_ESM.docx]

**Additional file 5: Table S2** Attention items in this procedure.

| **Step** | **Attention** |
| --- | --- |
| 1A(ii) | If *Global* is checked in the *Set scale* window, a window will appear when more images are imported into FIJI. Uncheck *Disable Global Calibration* to enable the same scale for newly imported images. |
| 1A(iii) | The clarity of the images can also be improved by using subtract background, smooth, and sharpen. In the *Process* list, click *Subtract Background* (*Rolling ball radius* can be adjusted from 20~80 depend on the image background), *Smooth*, or *Sharpen* to remove the background noise signal, smooth or sharp the image when needed. |
| 1A(iv) | If the cellular outline signal is not clear enough after threshold adjustment, please back to step 1A(iii) and improve image quality |
| 1A(v) | The balance of erode and dilate adjustment should pay careful attention, because *Process* > *Binary* > *Erode* can reduce the cell size and *Process* > *Binary* > *Dilate* might crack the cell outlines. |
| 1A(vi) | The ROI can be applied to select the cells, which should be identified when needed. In the *Analyze* list, choose *Tools* > *ROI Manager* to open the ROI manager window. First, select the *Polygon*/*Freehand selection* button from the FIJI panel and draw an ROI on the image, and then click *Add [t]* button in *the ROI manager window* to record the ROI. At last, progress particle analysis |
| 1A(vii) | The maximum or minimum cells can be found in the image and the size is displayed in the result list with the same number to the particle nearby on the image |
| 1B(iii) | The figure can be exported as the format of .pdf or some other vectogram for making further edits in the software of AI |
| 2A(ii) | The following analysis does not need the *intensity* and *frame* information because the centroid coordinate data are not super-resolution fluorescence signal, the *frame* could be any other numbers but all the *intensity* column and the *frame* column cannot miss. |
| 2B(iv) | The size of the dots can be adjusted by inputting the number in the *size point* textbox. Select the *glass* icon or scrolling the mouse wheel for zoom adjustment. Select the *grid* icon to display a grid corresponding to a low-resolution pixel size. The *hand* icon can scroll the image. |
| 2B(v) | Outline of the Voronoï polygons can be displayed by unchecking the *Fill polygon* checkbox. The histogram diagram can be better displayed (depend on the distribution of the cell location) by checking the *Log scale* checkbox under the histogram diagram |
| 2B(viii) | The density factor can also be modulated under the *Voronoï construction* tab by adjusting the histogram diagram (left and right click for defining the minimum and maximum value respectively). Object shape and outline can be displayed by checking the *Display shape* and *Display outline* checkboxes respectively. The object's creation is also determined by the selection of L*ocal Densities*, *Mean Distance*, and *Area*. |
| 2B(ix) | The exported graph can be altered to various combinations by checking or unchecking *Display* checkboxes under the *Filters* tab to show or hide point, by checking or unchecking *Display shape* or *Display outline* checkboxes under *VoronoÏ diagram* tab to show or hide object shape and outline, and by checking or unchecking *Fill polygon* checkbox to fill the Voronoï polygons. |
| 2C(ii) | Turn down the transparency of origin image appropriately and to make Voronoï diagram more clarity |
| 2C(iii) | The figure can be exported according to the user-defined board or preinstall size of the board which can be chosen by checking *a Use artboards text box* or not when exporting the images. |
