# Supplementary material for: High-efficiency procedure to characterize, segment, and quantify complex multicellularity in raw micrographs in plants
Source: Plant Methods. 2020 Jul 28;16:100. doi: 10.1186/s13007-020-00642-0 (PMC7390866; doi:10.1186/s13007-020-00642-0)
Supplement: Supplementary file 6 — Additional file 6: Table S3. Troubleshooting in this procedure. [file 13007_2020_642_MOESM6_ESM.docx]

**Additional file 6: Table S3** Troubleshooting in this procedure.

| **Step** | **Problem** | **Possible reason** | **Solution** |
| --- | --- | --- | --- |
| 1A(iv) | The binary signal of cell outline shows noise dots and are not continuous lines | The resolution of the image is low | Upgrading the resolution by clicking *Image > Adjust > Size* and inputting *Width*/*Height* (*pixels*) |
| 1A(vi) | The recognition results are not based on ROI | The ROI does not add to the *ROI manager* window | Click *Add [t]* button in *the ROI manager window* to record the ROI before progressing particle analysis |
|  | No particles were detected | The assumed threshold (255-255) may not be correct | Readjustment the threshold of the image and ensure the image should be binarization |
|  |  | The size setup may not be correct | Readjustment the size in *Analyze Particles* windows. |
| 1A(vii) | The result list contains too many small particles which should be background signal or noise point | The resolution of the image is low or inappropriate size value setting of particles | Follow Table 1 step 1A(iv) or define the size range of particles |
| 2B(ii) | An error displayed in the software when an image is imported | The data format may be incorrect | The centroid data should be .csv format. Check the data again and ensure the data are strictly following the layout of Table 2 |
| 2B(vii) | No clusters are identified | Object creation may be too small | Increase the range of Object when object created |
|  |  | The *Density factor* may not correct | Readjustment the *Density factor* under *Clusters definition* of *Clusters tab* |
| 2C(iii) | The image exported cannot edit in AI software | The format of the image exported is incorrect | Correct the format of the image to .svg when exporting the images |
